# Supplementary material for: Scoping review protocol: is there a role for physical activity interventions in the treatment pathway of bladder cancer?
Source: BMJ Open. 2019 Nov 12;9(11):e033518. doi: 10.1136/bmjopen-2019-033518 (PMC6858121; doi:10.1136/bmjopen-2019-033518)
Supplement: Supplementary data [file bmjopen-2019-033518supp001.pdf]

Appendix – search strategy for Medline (PubMed) and Ovid Gateway (Embase and Ovid)

Bladder cancer

exp exercise/ or exp anaerobic exercise/ or exp aerobic exercise/ or exp isotonic exercise/ or exp isokinetic exercise/ or exp isometric exercise/

Bladder cancer.tw.

Exercise.tw.

Exp physical activity/

Exercise program$.tw.

Exp bladder tumor/ or exp bladder cancer/ or exp bladder carcinoma/ or exp bladder cancer tumour

vesical cancer.tw.

Vesical neoplasm.tw.

Urinary Bladder Neoplasms OR Bladder Carcinoma OR bladder malignancy OR bladder neoplasm OR bladder malignancies OR cystectomy or cystourethrectomy . mp
