# Supplementary material for: Scoping review protocol: is there a role for physical activity interventions in the treatment pathway of bladder cancer?
Source: BMJ Open. 2019 Nov 12;9(11):e033518. doi: 10.1136/bmjopen-2019-033518 (PMC6858121; doi:10.1136/bmjopen-2019-033518)
Supplement: Author's manuscript [file bmjopen-2019-033518.draft_revisions.pdf]

BMJ Open

BMJ Open is committed to open peer review. As part of this commitment we make the peer review history of every article we publish publicly available.

When an article is published we post the peer reviewers' comments and the authors' responses online. We also post the versions of the paper that were used during peer review. These are the versions that the peer review comments apply to.

The versions of the paper that follow are the versions that were submitted during the peer review process. They are not the versions of record or the final published versions. They should not be cited or distributed as the published version of this manuscript.

BMJ Open is an open access journal and the full, final, typeset and author-corrected version of record of the manuscript is available on our site with no access controls, subscription charges or pay-per-view fees (http://bmjopen.bmj.com).

If you have any questions on BMJ Open's open peer review process please email info.bmjopen@bmj.com

BMJ Open

Scoping Review: Is there a role for physical activity interventions in the treatment pathway of bladder cancer?

Journal:	*BMJ Open*
Manuscript ID	bmjopen-2019-033518
Article Type:	Protocol
Date Submitted by the Author:	08-Aug-2019

Complete List of Authors:	Mehrotra, Sneha; King's College London, Translational Oncology and Urology Research<br>Rowland, Megan<br>Zhang, Hanyu<br>Russell, Beth ; King's College London, School of Cancer and Pharmaceutical Studies, Translational Oncology & Urology Research (TOUR)<br>Fox, Louis; King's College London, Translational Oncology and Urology Research<br>Beyer, Katharina<br>Rammant, Elke<br>Peat, Nicola<br>Van Hemelrijck, Mieke; Kings College London, Translational Oncology & Urology Offices<br>Bosco, Cecilia; King's College London School of Medical Education,

Keywords:	bladder cancer, exercise, physical activity, quality of life

SCHOLARONE™ Manuscripts

Scoping Review: Is there a role for physical activity interventions in the treatment pathway of bladder cancer?

Sneha Mehrotra[1], Megan Rowland[1], Hanyu Zhang[1], Beth Russell[1], Louis Fox[1], Katharina Beyer[1], Elke Rammant[3], Nicola Peat[2], Mieke Van Hemelrijck[1], Cecilia Bosco[1].

Authors Affiliations:

1. King's College London, Faculty of Life Sciences and Medicine, Translational Oncology and Urology Research, London, UK
2. Guy's and St Thomas' NHS Foundation Trust, Comprehensive Cancer Centre, Department of Physiotherapy, London, UK
3. Ghent University, Department of Human Structure and Repair, Ghent, BE

Corresponding author and contact details:

Mieke Van Hemelrijck
King's College London
Translational Oncology and Urology Research
3rd Floor Bermondsey Wing, Guy's Hospital
London
SE1 9RT
United Kingdom
Mieke.vanhemelrijck@kcl.ac.uk

Key words: Bladder cancer, physical activity interventions, scoping review

Word count: 250 (abstract) and 1,687 (protocol)

Conflicts of interest: None to be declared.

Author contribution:
Design of protocol: SM, MR, HZ, BR, LF, KB, MVH, CB
Draft of manuscript: SM, MR, HZ, CB
Final approval of manuscript: SM, MR, HZ, BR, LF, KB, ER, NP, MVH, CB

Funding: None to be declared.

Acknowledgement: The authors gratefully acknowledge the helpful comments from the bladder cancer patient support group.

For peer review only

Abstract

Introduction: Bladder cancer (BC) patients have been found to have worse cancer experiences than those with other cancers – which may partly be due to impact on quality of life. Currently, little is known about the impact of physical activity (PA) on BC outcomes. This scoping review aims to identify what associations have been found with lifestyle PA or PA interventions (including their efficacy and feasibility), as well as the potential underlying biological mechanisms for their effects.

Methods and analysis: PRISM ScR and Levac et al. methodology frameworks will be used to complete this scoping review. Electronic databases will be searched (MEDLINE, EMBASE, the Cochrane Library, grey literature sources). Two independent reviewers will screen all abstracts and titles and during a second stage and full-text publications for inclusion. All studies describing PA (as an existing lifestyle or as part of an intervention programme), during BC management will be included. Study characteristics will be recorded; qualitative and quantitative data will be extracted and summarised. A further consultation step will be carried out with patients, their family members and healthcare professionals.

Ethics and dissemination: Results will be disseminated through a peer-reviewed publication. Through the consultation step, we will ensure that findings will reach a wide audience and recommendations can be made for future development of PA interventions for BC patients. Data used will be from publicly available secondary sources and the consultation step will be carried out as part of Patient and Public Involvement (PPI), so this study does not require ethical review.

For peer review only - http://bmjopen.bmj.com/site/about/guidelines.xhtml

Strengths and limitations of this study

- This is a novel scoping review to understand how physical activity affects management of bladder cancer.
- This review will extend the current reviews as it focuses on all bladder cancer patients, both observational and randomised trial data, and assesses evidence for potential underlying biological mechanisms.
- Stakeholders including patients, their family members, urologists, oncologists, physiotherapists will be involved throughout the study.
- The identification and synthesis of data will also cover the grey literature – which is not as easily searchable as the peer-reviewed published literature.
- It is possible that our review will not include all articles which have been published in every journal as some may not be accessible.

For peer review only

For peer review only - http://bmjopen.bmj.com/site/about/guidelines.xhtml

Introduction

Bladder cancer (BC) is the 9th most common malignancy worldwide. There are approximately 10,300 new BC cases diagnosed in the UK every year, which equates to about 28 new diagnoses every day (1). BC accounts for 3% of all new cancer cases, and is the 8th most common male and 16th most common female cancer (2).

The 2015 NICE guidelines for BC diagnosis and management stated (3): “There is thought to be considerable variation across the NHS in diagnosis and management of BC and provision of care to people who have it. There is evidence that the patient experience for people with BC is worse than that for people with other cancer.” The cause of the poor patient experience is multifactorial – but is mainly driven by the effects of cancer diagnosis and treatment on health-related quality of life (HRQoL)(4). About 30% of patients present with muscle-invasive BC (MIBC), although up to 45% of those presenting with non-muscle-invasive BC (NMBC) will subsequently progress to MIBC. Treatments for NMBC and MIBC differ, but both come with a variety of side-effects, as well as invasive investigations and frequent follow-up due to the risk of recurrence and metastasis (5).

Despite the burden of BC on different HRQoL domains (4), relatively few HRQoL studies have been conducted in patients with BC compared to other tumour groups – so that it is also unclear how this may differ between those with NMBC and MIBC (6, 7). A contributing factor for this lack of knowledge is the small investment in BC research: in the UK, the total annual research spend on BC is only £216 per new patient - compared to £561 for prostate cancer (8).

For peer review only - http://bmjopen.bmj.com/site/about/guidelines.xhtml

Study rationale

Physical activity (PA) interventions have been introduced for cancer patients as they are thought to contribute to better treatment outcomes and increase HRQoL (9). More specifically, it has been suggested that integrating exercise training with standard cancer care and treatment may improve disease-related physiological and psychological outcomes in patients, as it helps to reduce drug toxicities and increases treatment completion rates (10). For example, an analysis by Holmes et al. in the Nurses' Health Study highlighted that engagement in more than 9 MET h/week of PA following breast-cancer diagnosis was inversely associated with breast cancer-specific mortality risk (11). A recent systematic review showed that the greater intensity PA interventions were associated with a greater beneficial effect on HRQoL - which included the assessment of factors such as social functioning and fatigue (12). Several mechanisms such as enhanced immune response, body composition, tumour vascularisation and tryptophan metabolism regulation have been suggested to explain the potential benefits of PA interventions in cancer patients (10).

In contrast to some other cancers, the literature on the potential positive effects of PA on BC management has not been addressed comprehensively across the treatment pathway – with currently one review focusing specifically on those patients who have undergone a radical cystectomy, which is only affecting a subgroup of BC patients (13).

Study objectives

With this scoping review, we aim to investigate what information is currently available on the effects of PA on the various stages of the BC treatment pathway. This will help identify the gaps in the current work on the influence of PA on BC management. We will describe

For peer review only - http://bmjopen.bmj.com/site/about/guidelines.xhtml

types and duration of PA (as an existing lifestyle or as part of an intervention programme), the reported effects on clinical outcomes and HRQoL, the feasibility of PA interventions, and the proposed biological mechanisms (as this may also inform the target population and PA intervention design). Data from both observational and randomised studies will ultimately help us design a PA intervention which is beneficial for the BC patients, but also acceptable and feasible for all stakeholders.

Protocol design

Methods for this scoping review were developed based on the Joanna Briggs Institute guidelines (14) and more specifically the guidelines developed by Levac *et al.* (15), which describe the below six framework stages.

Stage 1: Identifying the research question

Through consultation with the clinical research team, the overall research questions are defined as:

1. What type of PA (lifestyle measurements or PA interventions) has currently been reported to affect clinical outcomes and/or HRQoL for BC patients?
2. What effects of PA (interventions) on clinical outcomes and HRQoL for BC have been reported?
3. What information is available on the feasibility of existing PA interventions?
4. Is there evidence to support underlying biological mechanisms for the effect of PA on BC development/outcomes?

Stage 2: Identifying relevant studies - Search strategy

For peer review only - http://bmjopen.bmj.com/site/about/guidelines.xhtml

The following electronic databases will be searched from inception until the date in which the searches will be performed: MEDLINE (using the PubMed interface) and Ovid Gateway (Embase and PsycInfo and Health). The search strategies will be evaluated using the Peer Review of Electronic Search Strategies (PRESS) guidelines (16).

Search terms have been determined through researcher input and researching the current available literature to help guide the selection of terms – ensuring they are broad enough to capture any PA intervention and BC study. Our scoping review will analyse both quantitative and qualitative data on PA and BC (see search strategy in Appendix). To ensure that all relevant information is retrieved, relevant grey literature sources will be searched.

Stage 3: Study selection

The studies that will be included will be determined through the use of the PRISMA-ScR extension for scoping reviews (17). Studies will be considered for inclusion if they assess clinical/HRQoL outcomes in patients who have a primary diagnosis of BCa and physical activity has been measured (either as part of their lifestyle or as part of an intervention in the treatment pathway). Hence, we will include both observational studies and randomised controlled trials. Studies will be excluded if the publication is not available in English. All papers derived from the organ digital search process will be uploaded to a reference management software (Endnote). From these references, we will then document the exclusion process of the studies; initially excluding irrelevant studies based on title alone, then based on abstracts. Two review authors will screen the studies independently - any lack of consensus will be discussed with a third review author. After screening titles and abstracts, the full articles will be read and considered for the review; those excluded will

For peer review only - http://bmjopen.bmj.com/site/about/guidelines.xhtml

have recorded evidence as to why this was necessary. For studies that have multiple publications of the same outcome(s) reported, the one with the longest follow-up will be selected. If older publications refer to articles, those included may be accessed to clarify methods if needed.

Stage 4: Charting the data

Two independent reviewers will conduct this process. The data extraction table produced will include at least the following headings:

a. Author
b. Year of publication
c. Country where the study was published/conducted
d. Aims/purpose
e. Study population and study size
f. Study design (e.g. observational, RCT)
g. PA type + details (e.g. lifestyle, intervention)
h. Duration of intervention
i. Outcomes
j. Key findings that relate to scoping review objectives

Stage 5: Collating, summarizing, and reporting the results

For our scoping review, the studies identified will be analysed using both qualitative and quantitative methods. An overview of the research will be displayed through all the findings.

For peer review only - http://bmjopen.bmj.com/site/about/guidelines.xhtml

In terms of qualitative aspects, all the reported insights will be deductively coded into a conceptual model that is adapted from the Donabedian conceptual framework to determine the advantages of PA for BC patients (18). This framework specifically will be used to assess the quality of care and thus the effectiveness of the PA interventions reported through three domains: structure, process and outcome. By assessing interventions through three different domains, we will be able to identify the different strengths of PA interventions and use this to guide the design of future feasibility trials. *Structure* will regard the organisation, resources and equipment available and needed for the interventions. *Process* will evaluate how the intervention is implemented and the methods used by healthcare professionals when delivering the intervention and the effect this has on the patient's HRQoL. *Outcome* will address the overall mental state and feelings of patients taking part in the interventions (18).

The quantitative outcomes will be assessed using information on the following measurements:

- Survival or recurrence rates/response to treatment/HRQoL measures: to inform on efficacy of the PA lifestyle/intervention as well as patient selection
- Adherence to the intervention: indication of practicality, acceptability and feasibility of PA interventions

Stage 6: Consultation – Patient and Public Involvement

This scoping review is a first phase in a multistage research programme (19) aimed at developing a feasibility PA intervention for patients with BC. To ensure that our assessment of the existing evidence for the implementation of PA interventions in the BC treatment pathway identifies the right target population and format/timing of a PA intervention, we

For peer review only - http://bmjopen.bmj.com/site/about/guidelines.xhtml

also aim to include a consultation phase in this scoping review. The results from this scoping review combined with the consultation phase will then lead to development of a PA intervention that it can be implemented in standard care.

This consultation phase is part of our Patient and Public Involvement strategy as we will work actively in partnership with patients, their family members, and healthcare professionals to plan and design future PA interventions for BC patients (20). More specifically, we will run focus groups with BC patients and their families, healthcare professionals, physiotherapists, and behavioural scientist to help identify whether the results of the scoping review truly reflect the needs and expectations of all stakeholders involved. We will invite and recruit participants for this consultation phase through our ongoing collaborations with patient advocacy organisations and healthcare professional organisations (e.g. Action Bladder Cancer UK, Fight Bladder Cancer, British Association of Urological Surgeons, British Uro-Oncology Group)(21).

Dissemination and ethics

As outlined above, this scoping review with a consultation phase will constitute the first stage in a multistage research programme aimed at developing a feasibility PA intervention for patients with BC.

As the scoping review methodology is based on reviewing and collecting data from publicly available materials, this study does not require ethics approval. To facilitate knowledge translation activities, we will liaise with relevant stakeholders through patient advocate and healthcare professional organisations.

For peer review only - http://bmjopen.bmj.com/site/about/guidelines.xhtml

References

1. Kockelbergh R, Hounsome LS, Mayer E. The Epidemiology of urological cancer 2001–2013. Journal of Clinical Urology. 2017;10:3-8.

2. Cancer Research UK. Bladder Cancer Statistics 2019 [Available from: https://www.cancerresearchuk.org/health-professional/cancer-statistics/statistics-by-cancer-type/bladder-cancer#heading-Zero.

3. National Institute for health and Care Excellence. Bladder cancer: diagnosis and management. 2019 28 June.

4. Smith AB, Jaeger B, Pinheiro LC, Edwards LJ, Tan HJ, Nielsen ME, et al. Impact of bladder cancer on health-related quality of life. BJU Int. 2018;121(4):549-57.

5. van Rhijn BW, Burger M, Lotan Y, Solsona E, Stief CG, Sylvester RJ, et al. Recurrence and progression of disease in non–muscle-invasive bladder cancer: from epidemiology to treatment strategy. European urology. 2009;56(3):430-42.

6. Singer S, Ziegler C, Schwalenberg T, Hinz A, Götze H, Schulte T. Quality of life in patients with muscle invasive and non-muscle invasive bladder cancer. Supportive Care in Cancer. 2013;21(5):1383-93.

7. Yu EY, Nekeman D, Aaronson NK, Billingham LJ, James ND, Cheng KK, et al. Health-Related Quality of Life in Bladder Cancer Patients around the Time of Diagnosis. BJU Int. 2019.

8. National Cancer Research Institute. Spend by Research Category and Disease Site. [Date accessed: 14 November 2018]. Available from: https://www.ncri.org.uk/ncri-cancer-research-database/spend-by-research-category-and-disease-site/.

9. Albrecht TA, Taylor AG. Physical activity in patients with advanced-stage cancer: a systematic review of the literature. Clin J Oncol Nurs. 2012;16(3):293-300.

10. Christensen JF, Simonsen C, Hojman P. Exercise Training in Cancer Control and Treatment. Compr Physiol. 2018;9(1):165-205.

11. Holmes MD, Chen WY, Feskanich D, Kroenke CH, Colditz GA. Physical Activity and Survival After Breast Cancer Diagnosis. JAMA. 2005;293(20):2479-86.

12. Mishra SI, Scherer RW, Geigle PM, Berlanstein DR, Topaloglu O, Gotay CC, et al. Exercise interventions on health-related quality of life for cancer survivors. Cochrane Database Syst Rev. 2012(8):CD007566.

13. Rammant E, Decaestecker K, Bultijnck R, Sundahl N, Ost P, Pauwels NS, et al. A systematic review of exercise and psychosocial rehabilitation interventions to improve

For peer review only - http://bmjopen.bmj.com/site/about/guidelines.xhtml

health-related outcomes in patients with bladder cancer undergoing radical cystectomy. Clin Rehabil. 2018;32(5):594-606.

14. Peters M, Godfrey C, McInerney P, Baldini Soares C, Khalil H, Parker D. Chapter 11: Scoping Reviews. In: In: Aromataris E MZE, editor. Joanna Briggs Institute Reviewer's Manual The Joanna Briggs Institute; 2017

15. Levac D, Colquhoun H, O'Brien KK. Scoping studies: advancing the methodology. Implementation science : IS. 2010;5:69-.

16. McGowan J, Sampson M, Salzwedel DM, Cogo E, Foerster V, Lefebvre C. PRESS Peer Review of Electronic Search Strategies: 2015 Guideline Statement. Journal of clinical epidemiology. 2016;75:40-6.

17. Tricco AC, Lillie E, Zarin W, O'Brien KK, Colquhoun H, Levac D, et al. PRISMA Extension for Scoping Reviews (PRISMA-ScR): Checklist and ExplanationThe PRISMA-ScR Statement. Annals of Internal Medicine. 2018;169(7):467-73.

18. Donabedian A. Explorations in quality assessment and monitoring: Health Administration Press; 1980.

19. Bleijenberg N, de Man-van Ginkel JM, Trappenburg JCA, Ettema RGA, Sino CG, Heim N, et al. Increasing value and reducing waste by optimizing the development of complex interventions: Enriching the development phase of the Medical Research Council (MRC) Framework. Int J Nurs Stud. 2018;79:86-93.

20. Baxter S, Clowes M, Muir D, Baird W, Broadway-Parkinson A, Bennett C. Supporting public involvement in interview and other panels: a systematic review. Health Expect. 2017;20(5):807-17.

21. Bessa A, Maclennan S, Enting D, Bryan RT, Josephs D, Hughes S, et al. Consensus in bladder cancer research priorities between patients and healthcare professionals using a four-stage modified Delphi method. Eur Urol. 2019;In press(

).

For peer review only

For peer review only - http://bmjopen.bmj.com/site/about/guidelines.xhtml

Appendix – search strategy

Bladder cancer

exp exercise/ or exp anaerobic exercise/ or exp aerobic exercise/ or exp isotonic exercise/ or exp isokinetic exercise/ or exp isometric exercise/

Bladder cancer.tw.

Exercise.tw.

Exp physical activity/

Exercise program$.tw.

Exp bladder tumor/ or exp bladder cancer/ or exp bladder carcinoma/ or exp bladder cancer tumour

vesical cancer.tw.

Vesical neoplasm.tw.

Urinary Bladder Neoplasms OR Bladder Carcinoma OR bladder malignancy OR bladder neoplasm OR bladder malignancies OR cystectomy or cystourethrectomy . mp

For peer review only

For peer review only - http://bmjopen.bmj.com/site/about/guidelines.xhtml

BMJ Open

Scoping Review Protocol: Is there a role for physical activity interventions in the treatment pathway of bladder cancer?

Journal:	*BMJ Open*
Manuscript ID	bmjopen-2019-033518.R1
Article Type:	Protocol
Date Submitted by the Author:	23-Oct-2019
Complete List of Authors:	Mehrotra, Sneha; King's College London, Translational Oncology and Urology Research<br>Rowland, Megan<br>Zhang, Hanyu<br>Russell, Beth ; King’s College London, School of Cancer and Pharmaceutical Studies, Translational Oncology & Urology Research (TOUR)<br>Fox, Louis; King's College London, Translational Oncology and Urology Research<br>Beyer, Katharina; King's College London, TOUR<br>Rammant, Elke ; Ghent University, Human structure and repair<br>Peat, Nicola; Guy's and St Thomas' NHS Foundation Trust, Physiotherapy Dept - Guy's Cancer Centre<br>Van Hemelrijck, Mieke; Kings College London, Translational Oncology & Urology Offices<br>Bosco, Cecilia; King's College London School of Medical Education,
**Primary Subject Heading**:	Epidemiology
Secondary Subject Heading:	Oncology
Keywords:	bladder cancer, exercise, physical activity, quality of life

SCHOLARONE™
Manuscripts

Scoping Review protocol: Is there a role for physical activity interventions in the treatment pathway of bladder cancer?

Sneha Mehrotra[1], Megan Rowland[1], Hanyu Zhang[1], Beth Russell[1], Louis Fox[1], Katharina Beyer[1], Elke Rammant[3], Nicola Peat[2], Mieke Van Hemelrijck[1], Cecilia Bosco[1].

Authors Affiliations:

1. King's College London, Faculty of Life Sciences and Medicine, Translational Oncology and Urology Research, London, UK
2. Guy's and St Thomas' NHS Foundation Trust, Comprehensive Cancer Centre, Department of Physiotherapy, London, UK
3. Ghent University, Department of Human Structure and Repair, Ghent, BE

Corresponding author and contact details:

Mieke Van Hemelrijck
King's College London
Translational Oncology and Urology Research
3rd Floor Bermondsey Wing, Guy's Hospital
London
SE1 9RT
United Kingdom
Mieke.vanhemelrijck@kcl.ac.uk

Key words: Bladder cancer, physical activity interventions, scoping review

Word count: 245 (abstract) and 1,687 (protocol)

Conflicts of interest: None to be declared.

Author contribution:
Design of protocol: SM, MR, HZ, BR, LF, KB, MVH, CB
Draft of manuscript: SM, MR, HZ, CB
Final approval of manuscript: SM, MR, HZ, BR, LF, KB, ER, NP, MVH, CB

Funding: We thank CRUK for their support (C45074/A26553).

Acknowledgement: The authors gratefully acknowledge the helpful comments from the bladder cancer patient support group.

For peer review only

For peer review only - http://bmjopen.bmj.com/site/about/guidelines.xhtml

Abstract

Introduction: Bladder cancer (BC) patients have been found to have worse experiences than those with other cancers – which may partly be due to impact on quality of life. Currently, little is known about the impact of physical activity (PA) on BC outcomes. This scoping review aims to identify what interventions are available, their reported efficacy and feasibility, and a description of potential underlying biological mechanisms for their effects.

Methods and analysis: PRISMA ScR guidelines and the Levac methodology framework will be followed/used. Electronic databases will be searched (MEDLINE, EMBASE, the Cochrane Library, PsycInfo and Health, OpenGray). Two independent reviewers will screen all abstracts and titles and during a second stage and full-text publications for inclusion. All studies describing PA (as an existing lifestyle or as part of an intervention programme), during BC management will be included. Study characteristics will be recorded; qualitative data will be extracted and evaluated using the Donabedian framework. Quantitative data will be extracted and summarised. A further consultation step will be carried out with patients, their family members and healthcare professionals.

Ethics and dissemination: Results will be disseminated through a peer-reviewed publication. Through the consultation step, we will ensure that findings will reach a wide audience and recommendations can be made for future development of PA interventions for BC patients. Data used will be from publicly available secondary sources and the consultation step will be carried out as part of patient and public involvement so this study does not require ethical review.

For peer review only - http://bmjopen.bmj.com/site/about/guidelines.xhtml

Strengths and limitations of this study

- This is a novel scoping review to understand what types of PA interventions exist within the management of bladder cancer.
- This review will extend the current reviews as it focuses on all bladder cancer patients, both observational and randomised trial data, and assesses evidence for potential underlying biological mechanisms.
- Stakeholders including patients, their family members, urologists, oncologists, physiotherapists will be involved throughout the study.
- The identification and synthesis of data will also cover the grey literature.
- It is possible that our review will not include all articles which have been published in every journal as some may not be accessible.

For peer review only

For peer review only - http://bmjopen.bmj.com/site/about/guidelines.xhtml

Introduction

Bladder cancer (BC) is the 9th most common malignancy worldwide. There are approximately 10,300 new BC cases diagnosed in the UK every year, which equates to about 28 new diagnoses every day (1). BC accounts for 3% of all new cancer cases, and is the 8th most common male and 16th most common female cancer (2).

The 2015 NICE guidelines for BC diagnosis and management stated (3): "There is thought to be considerable variation across the NHS in diagnosis and management of BC and provision of care to people who have it. There is evidence that the patient experience for people with BC is worse than that for people with other cancer." The cause of the poor patient experience is multifactorial – but is mainly driven by the effects of cancer diagnosis and treatment on health-related quality of life (HRQoL)(4). About 30% of patients present with muscle-invasive BC (MIBC), although up to 45% of those presenting with non-muscle-invasive BC (NMBC) will subsequently progress to MIBC. Treatments for NMBC and MIBC differ, but both come with a variety of side-effects, as well as invasive investigations and frequent follow-up due to the risk of recurrence and metastasis (5).

Despite the burden of BC on different HRQoL domains (4), relatively few HRQoL studies have been conducted in patients with BC compared to other tumour groups – so that it is also unclear how this may differ between those with NMBC and MIBC (6, 7). A contributing factor for this lack of knowledge is the small investment in BC research: in the UK, the total annual research spend on BC is only £216 per new patient - compared to £561 for prostate cancer (8).

For peer review only - http://bmjopen.bmj.com/site/about/guidelines.xhtml

Study rationale

Physical activity (PA) interventions have been introduced for cancer patients as they are thought to contribute to better treatment outcomes and increase HRQoL (9). More specifically, it has been suggested that integrating exercise training with standard cancer care and treatment may improve disease-related physiological and psychological outcomes in patients, as it helps to reduce drug toxicities and increases treatment completion rates (10). For example, an analysis by Holmes et al. in the Nurses' Health Study highlighted that engagement in more than 9 MET h/week of PA following breast-cancer diagnosis was inversely associated with breast cancer-specific mortality risk (11). A recent systematic review showed that the greater intensity PA interventions were associated with a greater beneficial effect on HRQoL - which included the assessment of factors such as social functioning and fatigue (12). Several mechanisms such as enhanced immune response, body composition, tumour vascularisation and tryptophan metabolism regulation have been suggested to explain the potential benefits of PA interventions in cancer patients (10).

In contrast to some other cancers, the literature on the potential positive effects of PA on BC management has not been addressed comprehensively across the treatment pathway – with currently one review focusing specifically on those patients who have undergone a radical cystectomy, which is only affecting a subgroup of BC patients (13).

Study objectives

With this scoping review, we aim to investigate what information is currently available on the effects of PA on the various stages of the BC treatment pathway. This will help identify the gaps in the current work on the influence of PA on BC management. We will describe

For peer review only - http://bmjopen.bmj.com/site/about/guidelines.xhtml

types and duration of PA (as an existing lifestyle or as part of an intervention programme), the reported effects on clinical outcomes and HRQoL, the feasibility of PA interventions, and describe the proposed biological mechanisms (as this may also inform the target population and PA intervention design). Data from all types of available studies - mainly observational and randomised studies - will ultimately help us design a PA intervention which is beneficial for BC patients, but also acceptable and feasible for all stakeholders.

Protocol design

Methods for this scoping review were developed based on the Joanna Briggs Institute guidelines (14) and more specifically the methodological guidelines developed by Levac *et al.* (15), which describe the below six framework stages. PRISMA-ScR extension for scoping reviews (16) will be followed to ensure that all suggested items are reported.

Stage 1: Identifying the research question

Through consultation with the clinical research team, the overall research questions are defined as:

1. What type of PA (lifestyle measurements or PA interventions) has currently been reported to affect clinical outcomes and/or HRQoL for BC patients?
2. What effects of PA (interventions) on clinical outcomes and HRQoL for BC have been reported?
3. What information is available on the feasibility of existing PA interventions?
4. Is there evidence to support underlying biological mechanisms for the effect of PA on BC development/outcomes?

For peer review only - http://bmjopen.bmj.com/site/about/guidelines.xhtml

Stage 2: Identifying relevant studies - Search strategy

The following electronic databases will be searched from inception until the date in which the searches will be performed (until November 2019): MEDLINE (using the PubMed interface) and Ovid Gateway (Embase and Ovid). The Cochrane Library, and OpenGray will also be searched. The search strategies will be evaluated using the Peer Review of Electronic Search Strategies (PRESS) guidelines (17).

Search terms have been determined through researcher input and researching the current available literature to help guide the selection of terms – ensuring they are broad enough to capture any PA intervention and BC study. Our scoping review will analyse both quantitative and qualitative data on PA and BC (see search strategy in Appendix). To ensure that all relevant information is retrieved, relevant grey literature sources will be searched.

Stage 3: Study selection

Studies will be considered for inclusion if they assess clinical/HRQoL outcomes in patients who have a primary diagnosis of BCa and physical activity has been measured (either as part of their lifestyle or as part of an intervention in the treatment pathway). Studies will be excluded if the publication is not available in English. All papers derived from the digital search process will be uploaded to a reference management software (Endnote). From these references, we will then document the exclusion process of the studies; initially excluding irrelevant studies based on title alone, then based on abstracts. Two review authors will screen the studies independently - any lack of consensus will be discussed with a third review author. After screening titles and abstracts, the full articles will be read and considered for the review also by two independent reviewers; those articles excluded will

For peer review only - http://bmjopen.bmj.com/site/about/guidelines.xhtml

have recorded evidence as to why this was necessary. For studies that have multiple publications of the same outcome(s) reported, the one with the longest follow-up will be selected. If older publications refer to articles, those included may be accessed to clarify methods if needed.

Stage 4: Charting the data

Two independent reviewers will conduct this process. The data extraction table produced will include at least the following headings:

a. Author
b. Year of publication
c. Country where the study was published/conducted
d. Aims/purpose
e. Study population and study size
f. Study design (e.g. observational, RCT)
g. PA type + details (e.g. lifestyle, intervention)
h. Duration of intervention
i. Outcomes
j. Key findings that relate to scoping review objectives

Stage 5: Collating, summarizing, and reporting the results

For our scoping review, the studies identified will be analysed using both qualitative and quantitative methods. An overview of the research will be displayed through all the findings.

For peer review only - http://bmjopen.bmj.com/site/about/guidelines.xhtml

In terms of qualitative aspects, all the reported insights will be deductively coded into a conceptual model that is taken from the Donabedian conceptual framework to determine the advantages of PA for BC patients (18). This framework will be used to assess the quality of care and thus the effectiveness of the PA interventions reported through three domains: structure, process and outcome. By assessing interventions through three different domains, we will be able to identify the different strengths of PA interventions and use this to guide the design of future feasibility trials. *Structure* will regard the organisation, resources and equipment available and needed for the interventions. *Process* will evaluate how the intervention is implemented and the methods used by healthcare professionals when delivering the intervention and the effect this has on the patient's HRQoL. *Outcome* will address the overall mental state and feelings of patients taking part in the interventions (18).

The quantitative outcomes will be assessed using information on the following measurements:

- Survival or recurrence rates/response to treatment/HRQoL measures: to inform on efficacy of the PA lifestyle/intervention as well as patient selection
- Adherence to the intervention: indication of practicality, acceptability and feasibility of PA interventions

Given the nature of this scoping review, we will not be explicitly performing a risk of bias assessment as usually required for quantitative systematic reviews (16) because of the following four reasons: (1) We will not compare the clinical outcomes of any clinical studies, but only describe interventions used and their potential effects; (2) argumentative and qualitative data do not lend themselves to the risk of bias assessment common in

For peer review only - http://bmjopen.bmj.com/site/about/guidelines.xhtml

quantitative systematic reviews; (3) we are likely to include many different types of studies which evaluations would have to be type-specific; and (4) quality assessments are likely be very difficult to standardize between reviewers and which could potentially add unnecessary bias into our own analyses."

Stage 6: Consultation – Patient and Public Involvement

This scoping review is a first phase in a multistage research programme (19) aimed at developing a feasibility PA intervention for patients with BC. To ensure that our assessment of the existing evidence for the implementation of PA interventions in the BC treatment pathway identifies the right target population and format/timing of a PA intervention, we also aim to include a consultation phase in this scoping review. The results from this scoping review combined with the consultation phase will then lead to development of a PA intervention that it can be implemented in standard care.

This consultation phase is part of our Patient and Public Involvement strategy as we will work actively in partnership with patients, their family members, and healthcare professionals to plan and design future PA interventions for BC patients (20). More specifically, we will run focus groups with BC patients and their families, healthcare professionals, physiotherapists, and behavioural scientist to help identify whether the results of the scoping review truly reflect the needs and expectations of all stakeholders involved. We will invite and recruit participants for this consultation phase through our ongoing collaborations with patient advocacy organisations and healthcare professional organisations (e.g. Action Bladder Cancer UK, Fight Bladder Cancer, British Association of Urological Surgeons, British Uro-Oncology Group)(21).

For peer review only - http://bmjopen.bmj.com/site/about/guidelines.xhtml

Dissemination and ethics

As outlined above, this scoping review with a consultation phase will constitute the first stage in a multistage research programme aimed at developing a feasibility PA intervention for patients with BC. Results will be disseminated through a peer-reviewed publication. Through the consultation step, we will ensure that findings will reach a wide audience and recommendations can be made for future development of PA interventions for BC patients.

As the scoping review methodology is based on reviewing and collecting data from publicly available materials, this study does not require ethics approval. To facilitate knowledge translation activities, we will liaise with relevant stakeholders through patient advocate and healthcare professional organisations. This consultation step will be carried out as part of patient and public involvement, so this study does not require ethical review.

For peer review only

For peer review only - http://bmjopen.bmj.com/site/about/guidelines.xhtml

References

1. Kockelbergh R, Hounsome LS, Mayer E. The Epidemiology of urological cancer 2001–2013. Journal of Clinical Urology. 2017;10:3-8.

2. Cancer Research UK. Bladder Cancer Statistics 2019 [Available from: https://www.cancerresearchuk.org/health-professional/cancer-statistics/statistics-by-cancer-type/bladder-cancer#heading-Zero.

3. National Institute for health and Care Excellence. Bladder cancer: diagnosis and management. 2019 28 June.

4. Smith AB, Jaeger B, Pinheiro LC, Edwards LJ, Tan HJ, Nielsen ME, et al. Impact of bladder cancer on health-related quality of life. BJU Int. 2018;121(4):549-57.

5. van Rhijn BW, Burger M, Lotan Y, Solsona E, Stief CG, Sylvester RJ, et al. Recurrence and progression of disease in non–muscle-invasive bladder cancer: from epidemiology to treatment strategy. European urology. 2009;56(3):430-42.

6. Singer S, Ziegler C, Schwalenberg T, Hinz A, Götze H, Schulte T. Quality of life in patients with muscle invasive and non-muscle invasive bladder cancer. Supportive Care in Cancer. 2013;21(5):1383-93.

7. Yu EY, Nekeman D, Aaronson NK, Billingham LJ, James ND, Cheng KK, et al. Health-Related Quality of Life in Bladder Cancer Patients around the Time of Diagnosis. BJU Int. 2019.

8. National Cancer Research Institute. Spend by Research Category and Disease Site. [Date accessed: 14 November 2018]. Available from: https://www.ncri.org.uk/ncri-cancer-research-database/spend-by-research-category-and-disease-site/.

9. Albrecht TA, Taylor AG. Physical activity in patients with advanced-stage cancer: a systematic review of the literature. Clin J Oncol Nurs. 2012;16(3):293-300.

10. Christensen JF, Simonsen C, Hojman P. Exercise Training in Cancer Control and Treatment. Compr Physiol. 2018;9(1):165-205.

11. Holmes MD, Chen WY, Feskanich D, Kroenke CH, Colditz GA. Physical Activity and Survival After Breast Cancer Diagnosis. JAMA. 2005;293(20):2479-86.

12. Mishra SI, Scherer RW, Geigle PM, Berlanstein DR, Topaloglu O, Gotay CC, et al. Exercise interventions on health-related quality of life for cancer survivors. Cochrane Database Syst Rev. 2012(8):CD007566.

13. Rammant E, Decaestecker K, Bultijnck R, Sundahl N, Ost P, Pauwels NS, et al. A systematic review of exercise and psychosocial rehabilitation interventions to improve

For peer review only - http://bmjopen.bmj.com/site/about/guidelines.xhtml

health-related outcomes in patients with bladder cancer undergoing radical cystectomy. Clin Rehabil. 2018;32(5):594-606.

14. Peters M, Godfrey C, McInerney P, Baldini Soares C, Khalil H, Parker D. Chapter 11: Scoping Reviews. In: In: Aromataris E MZE, editor. Joanna Briggs Institute Reviewer's Manual The Joanna Briggs Institute; 2017

15. Levac D, Colquhoun H, O'Brien KK. Scoping studies: advancing the methodology. Implementation science : IS. 2010;5:69-.

16. Tricco AC, Lillie E, Zarin W, O'Brien KK, Colquhoun H, Levac D, et al. PRISMA Extension for Scoping Reviews (PRISMA-ScR): Checklist and ExplanationThe PRISMA-ScR Statement. Annals of Internal Medicine. 2018;169(7):467-73.

17. McGowan J, Sampson M, Salzwedel DM, Cogo E, Foerster V, Lefebvre C. PRESS Peer Review of Electronic Search Strategies: 2015 Guideline Statement. Journal of clinical epidemiology. 2016;75:40-6.

18. Donabedian A. Explorations in quality assessment and monitoring: Health Administration Press; 1980.

19. Bleijenberg N, de Man-van Ginkel JM, Trappenburg JCA, Ettema RGA, Sino CG, Heim N, et al. Increasing value and reducing waste by optimizing the development of complex interventions: Enriching the development phase of the Medical Research Council (MRC) Framework. Int J Nurs Stud. 2018;79:86-93.

20. Baxter S, Clowes M, Muir D, Baird W, Broadway-Parkinson A, Bennett C. Supporting public involvement in interview and other panels: a systematic review. Health Expect. 2017;20(5):807-17.

21. Bessa A, Maclennan S, Enting D, Bryan RT, Josephs D, Hughes S, et al. Consensus in bladder cancer research priorities between patients and healthcare professionals using a four-stage modified Delphi method. Eur Urol. 2019;In press(

).

For peer review only

For peer review only - http://bmjopen.bmj.com/site/about/guidelines.xhtml

For peer review only

Appendix – search strategy for Medline (PubMed) and Ovid Gateway (Embase and Ovid)

Bladder cancer

exp exercise/ or exp anaerobic exercise/ or exp aerobic exercise/ or exp isotonic exercise/ or exp isokinetic exercise/ or exp isometric exercise/

Bladder cancer.tw.

Exercise.tw.

Exp physical activity/

Exercise program$.tw.

Exp bladder tumor/ or exp bladder cancer/ or exp bladder carcinoma/ or exp bladder cancer tumour

vesical cancer.tw.

Vesical neoplasm.tw.

Urinary Bladder Neoplasms OR Bladder Carcinoma OR bladder malignancy OR bladder neoplasm OR bladder malignancies OR cystectomy or cystourethrectomy . mp

For peer review only
